# Les infections urinaires chez les patients insuffisants rénaux chroniques hospitalisés au service de néphrologie: profil bactériologique et facteurs de risque

**DOI:** 10.11604/pamj.2015.20.100.4356

**Published:** 2015-02-04

**Authors:** Abdeljalil Chemlal, Fatiha Alaoui Ismaili, Ilham Karimi, Ryme Elharraqui, Nawal Benabdellah, Samira Bekaoui, Intissar Haddiya, Yassamine Bentata

**Affiliations:** 1 Département de Néphrologie, Faculté de Médecine et de Pharmacie, Université Mohamed Premier, Oujda, Maroc

**Keywords:** Infection urinaire, insuffisance rénale chronique, bactériologie antibiothérapie, urinary tract infection, chronic renal failure, bacteriological antibiotherapy

## Abstract

L'infection urinaire chez l'insuffisant rénal est fréquente et particulière dans sa prise en charge diagnostique et thérapeutique. L'objectif de notre étude est de déterminer le profil bactériologique et d’étudier les facteurs de risque des infections urinaires chez le patient insuffisant rénal chronique en milieu de néphrologie. Etude prospective débutée en Septembre 2012 menée au service de néphrologie à l'hôpital régional d'Oujda de l'oriental Marocain. Ont été inclus tous les patients hospitalisés en néphrologie avec une infection urinaire documentée. Nous avons analysé les données démographiques, cliniques, biologiques, et thérapeutiques de nos patients à l'admission et au cours de leurs hospitalisations. 48 épisodes d'infections urinaires chez 43 patients ont été colligés dont 3 enfants. L'incidence de l'infection urinaire dans notre étude était de 4,65%. La médiane d’âge était de 53 (32-66) années. 60,4% étaient de sexe féminin. Le germe isolé était un Escheria Coli dans 58,3% et un Klebsiella dans 29,2%. Le germe isolé était résistant à l'amoxicilline-acide clavulanique dans 83% des cas. L'antibiotique prescris en premiére intention chez nos patients était une céphalosporine de 3 éme génération dans 50%. L’évolution chez nos patients était favorable dans 89,6% des cas. 33,3% avaient présenté un sepsis et on a noté le décès dans 10,4% des cas. Les infections urinaires chez l'insuffisant rénal chronique reste très grave vu leur lourde morbi-mortalié d'où l'intêrét d'un dépistage précoce chez cette population. Un usage raisonné des antibiotiques est nécessaire afin de prévenir l'extension des résistances bactériennes.

## Introduction

L′incidence annuelle des infections urinaires est estimée aux Etats-Unis à 11 millions de cas et en France à 4-6 millions de cas [1–3]. Au Maroc, les infections urinaires restent fréquentes et se situent en premier rang en milieu hospitalier. En néphrologie, l'infection urinaire constitue un motif fréquent de consultation et elle est présente chez un grand nombre de patients hospitalisés [4]. Très peu d’études sont disponibles sur ce sujet. L'infection urinaire chez l'insuffisant rénal est d'emblée une infection urinaire compliquée imposant une prise en charge diagnostique et thérapeutique particulière [5, 6]. C'est une pathologie fréquente mais grave car greffée d'une lourde morbi-mortalité [7]. Les prélèvements bactériologiques sont précieux, indispensables et permettent d′instaurer un traitement adapté. L'objectif de notre travail est de déterminer le profil bactériologique et d’étudier les facteurs de risque des infections urinaires chez le patient insuffisant rénal chronique en milieu de néphrologie.

## Méthodes

Nous avons réalisé une étude prospective débutée en Septembre 2012 menée au service de néphrologie à l'hôpital régional d'Oujda de l'oriental Marocain. Ont été inclus tous les patients hospitalisés au service de néphrologie, ayant une insuffisance rénale définie par un débit de filtration glomérulaire < 60 ml/min/m^2^ et/ou hémodialysés chroniques et présentant une infection urinaire documentée. Tous ces patients hospitalisés en néphrologie de septembre 2012 à janvier 2014 ont bénéficié d'un ECBU systématique. Ont été exclus les patients présentant une infection urinaire sans preuve bactériologique, une cystite simple ou une infection urinaire documentée chez un patient vu en consultation de néphrologie. Nous avons analysé les données démographiques, cliniques, biologiques, et thérapeutiques de nos patients à l'admission et au cours de leurs hospitalisations. La fonction rénale a été estimée par la formule MDRD (Modification of Diet Renal Disease) et la classification de l'insuffisance rénale répondait aux critères de KDOQI (Kidney Disease Outcomes Quality Initiative). L'analyse statistique a été effectuée par le logiciel SPSS 20.

## Résultats

48 épisodes d′infections urinaires chez 43 patients ont été colligés. 40 Adultes et 3 enfants. L'incidence de l'infection urinaire dans notre étude chez les patients hospitalisés quelque soit le DFG était de 4,65%. La médiane d′âge était de 53 (32-66) années. 60,4% étaient de sexe féminin. La médiane d'hospitalisation était de 11 [7–15] jours.16, 7%, 16,7% et 41,6% des patients avaient une insuffisance rénale stade 3,4 et 5 respectivement (selon la formule MDRD). 25% étaient des hémodialysés chroniques. 62,5% de nos patients étaient de bas niveau socioéconomique. Le diabète était noté dans 35,4%, l'hypertension artérielle dans 33,3% et la lithiase rénale dans 16,7% des cas. 25% ont bénéficié de la pose d'une sonde urinaire au cours de l'année précédant l’épisode actuel et 27,1% ont bénéficié d'une pose de sonde urinaire au cours de l'hospitalisation actuelle. (Le Tableau 1 rapporte les données démographiques et cliniques de nos patients).

**Tableau 1:** Caractéristiques démographiques et cliniques des patients

Caractéristiques (n = 48)	Résultats N (%)
**A L'admission**	
Niveau socio-économique bas	30 (62,5)
Niveau socio-économique moyen	17 (35,4)
**Degré d'insuffisance rénale**	
Stade 3	8 (16,7)
Stade 4	8 (16,7)
Stade 5	20 (41,60)
Hémodialysé chronique	12 ( 25%)
**Pathologies associées:**	
Diabète type 1	5 (10,4)
Diabète type 2	12 (25)
Lithiase rénale	8 (16,5)
Hypertension artérielle	16 (33,3)
**Antécédents:**	
Antécédent d'hospitalisation < 1an	19 (39,6)
Antécédent d'infection urinaire < 1 an	17 (35,4)
Antécédent de sonde urinaire < 1an	12 (25)
Antécédent de sonde JJ < 1 an	4 (8,3)

Le germe isolé était un Escheria Coli dans 58,3% et un Klebsiella dans 29,2%. Le candida albicans a été associé dans 4,2% des cas. 16,7% des bactéries étaient une BLSE. Le germe isolé était résistant à l'amoxicilline-acide clavulanique dans 83% des cas, au céphalosporine de 3 éme génération dans 25,5% et aux quinolones dans 29,8%. (Le Tableau 2 rapporte les résultats de l'examen cyto-bactériologique des urines et de l'antibiogramme). L'antibiotique prescris en premiére intention chez nos patients était une céphalosporine de 3 éme génération dans 50%, une ciprofloxacine dans 31,3% et un aminoside a été associé dans 22,9%. L’évolution chez nos patients était favorable dans 89,6% des cas. 33,3% avaient présenté un sepsis. Le recours à la dialyse était noté dans 52,1% et le décès dans 10,4% des cas. (Le Tableau 3 rapporte les données thérapeutiques et évolutives chez nos patients).

**Tableau 2:** Résultats de l'examen cyto-bactériologique des urines et de l'antibiogramme

Caractéristiques (n = 48)	Résultats N (%)
Urines troubles	48 (100)
**Germes**	
Escheria.Coli	28 (58,3)
Klebsiella	14 (29,2)
Streptocoque	2 (4,2)
Proteus sp	1 (2,1)
Staphylocoque saprophyticus	2 (4,2)
Candidas albicans associées	2 (4,2)
BLSE (bétalactamase à spectre élargie)	8 (16,7)
**Sensibilité à:**	
Amoxicilline-acide clavulanique	8 (16,7)
Céphalosporine 3ème génération	35 (72,9)
Ciprofloxacine	33 (68,8)

**Tableau 3:** Données thérapeutiques et évolutives chez nos patients

Traitement (n = 48)	Résultats N (%)
**Antibiotique prescrit**	
Amoxicilline-acide clavulanique	4 (8,3)
Céphalosporine 3ème génération	24 (50)
Ciprofloxacine	15 (31,3)
Colistine	1 (2,1)
Céftazidime	2 (4,2)
Imipénème	2 (4,2)
Aminosides associés	11 (22,9)
**Evolution**	
Admission par les Urgences	32 (66,7)
Séjour en réanimation lors de l'hospitalisation	5 (10,4)
Port d'une sonde urinaire au cours de l'hospitalisation	13 (27,1)
Sepsis associé	16 (33,3)
Recours à la dialyse	25 (52,1)
Décès	5 (10,4)

## Discussion

Notre étude a objectivé que l'incidence de l'infection urinaire en milieu de néphrologie est élevée. Elle est de l'ordre de 4,65% vs 9% dans une autre étude marocaine menée au service de néphrologie concernant tous les patients ayant ou pas une insuffisance rénale [4]. L’ infection urinaire était la premiére cause des infections nosocomiales selon les enquêtes de prévalence 2009-2010 faites dans 8 établissements et 63 services dont 8 de réanimation au CHU IBN SINA à Rabat au Maroc [8]. L'infection urinaire a fait l'objet de nombreuses études du fait de sa fréquence et sa gravité étiologique ou évolutive, motivant des prescriptions d'antibiotiques le plus souvent sans savoir le germe causal ni son profile vis-à-vis aux antibiotiques [9]. Dans ce contexte, l'infection urinaire est souvent d'emblée compliquée et nécessite un diagnostic précoce et précis et une prise en charge adaptée. Elle peut entraîner des complications d'ordre général, telle qu'une septicémie à point de départ urinaire [10].

La plupart des études épidémiologiques [11–18] ont démontré que l'infection urinaire est plus fréquente chez la femme que chez l'homme. Dans notre étude, on a noté une prédominance féminine de 60,4%. La nature des germes isolés à l'ECBU varie selon les études. Au CHU Ibn Rochd, lors d'une étude rétrospective portant sur 3933 ECBU provenant de divers services hospitaliers, ils ont observé la prédominance des entérobactéries avec une fréquence égale d'E. Coli et de Klebsiella [19]. Dans l’étude MAROCAINE, les germes les plus fréquemment retrouvés étaient des entérobactéries: 60% des cas, avec prédominance d'E. Coli, suivi de Klebsiella, Enterobacter et de Protéus [4]. Selon l’étude TUNISIENNE sur les infections urinaires hautes, les entérobactéries ont été isolées dans 93,5% des cas dont E. coli dans 73,3% et Klebsiella pneumoniae dans 15,3% [20].

Dans notre série, les entérobactéries ont repésenté 89% avec une prédominace d’ E. coli de 58%, suivi de Klebsiella, et de Protéus. L’émergence des germes urinaires multi résistants pose un grand problème en pratique quotidienne. Il est à l'origine de la prolongation de la durée d'hôspitalisation et en conséquent d'une morbidité plus importante. L’émergence de résistance étant intimement liée au contact antérieur avec les antibiotiques, aux hôspitalisations, aux antibiothérapies prolongées parfois non justifiées et à l'antibioprophylaxie abusive. La lutte contre ce phénomène passe par le respect des règles de prescription antibiotique, le respect des bactériuries asymptomatiques et la mise en place d'une politique de l'antibiothérapie [21–23]. La sensibilité aux antibiotiques des germes incriminés (surtout des entérobactéries) est en baisse constante de part le monde, mais avec des différences entre les séries. Nous avons comparé nos données à celles de la littérature afin de rechercher une différence entre les régions (Le Tableau 4 rapporte la résistance de l'E. coli aux antibiotiques selon différentes études) [24–27]. Les différences de sensibilité sont dues à la pression de sélection par l'usage trop fréquent du même antibiotique dans une région donnée.

**Tableau 4:** Résistance de E. Coli aux antibiotiques

Etude	Ampicilline	Céphalosporine 3 éme génération	Ciprofloxacine
Notre série	75	17,85	28,57
Tunisie (20)	68	1	9
Taiwan (24)	78	4	12
Turquie (25)	73,3	-	12
USA (26)	39,3	-	6,8
Russie (27)	37,1	-	4,5
Canada (26)	33	-	1,1

Les recommandations des bonnes pratiques pour la prise en charge des infections urinaires sont définies par les sociétés savantes. Cependant, le cas particulier du traitement de l'infection urinaire chez l'insuffisant rénal est rarement évoqué [28]. L'instauration d'une antibiothérapie qui représente la pierre angulaire du traitement impose une estimation précise de la fonction rénale pour adapter finement les posologies des antibiotiques afin d’éviter le risque de surdosage des antibiotiques et de survenue de complications [29, 30].

## Conclusion

L'infection urinaire est la pathologie infectieuse bactérienne la plus fréquente dans la population générale, y compris chez les patients avec une insuffisance rénale. L'enjeu principal chez les patients insuffisants rénaux est l'adaptation des posologies des traitements antibiotiques. Le meilleur traitement reste la prévention par le dépistage précoce des infections urinaires chez le patient insuffisant rénal et le respect des bonnes pratiques de prescription des antibiotiques. La maîtrise de la résistance bactérienne aux antibiotiques est une priorité de santé publique qui nécessite des actions concertées dans les établissements de santé et de recherche. La prévention de la transmission croisée et la réduction de la pression de sélection, par un usage rationnel des antibiotiques, en sont les deux composantes essentielles.
